# Transcriptome Analysis Reveal Candidate Genes and Pathways Responses to Lactate Dehydrogenase Inhibition (Oxamate) in Hyperglycemic Human Renal Proximal Epithelial Tubular Cells

**DOI:** 10.3389/fendo.2022.785605

**Published:** 2022-03-17

**Authors:** Zhimin Wang, Dan Hao, Dong Fang, Jiating Yu, Xiao Wang, Guijun Qin

**Affiliations:** ^1^Division of Endocrinology and Metabolic Diseases, The First Affiliated Hospital of Zhengzhou University, Zhengzhou, China; ^2^Shaanxi Key Laboratory of Animal Genetics, Breeding and Reproduction, College of Animal Science and Technology, Northwest A&F University, Yangling, China; ^3^Department of Urology, Peking University First Hospital, Institute of Urology, Peking University, Beijing, China; ^4^Division of Pediatric Surgery, The First Affiliated Hospital of Zhengzhou University, Zhengzhou, China; ^5^Konge Larsen ApS, Kongens Lyngby, Denmark

**Keywords:** diabetic kidney disease, HK-2 cells, high D-glucose, oxamate, transcriptomics, WGCNA, biological enrichment

## Abstract

Diabetic kidney disease (DKD) is the leading cause of both chronic kidney disease (CKD) and end-stage renal disease (ESRD). Previous studies showed that oxamate could regulate glycemic homeostasis and impacted mitochondria respiration in a hyperglycemia-dependent manner in the rat proximal tubular cells. To explore the transcriptome gene expression profiling of kidney tissues in human renal proximal epithelial tubular cell line (HK-2), we treated HK-2 cells with high D-glucose (HG) for 7 days before the addition of 40 mM oxamate for a further 24 hours in the presence of HG in this study. Afterwards, we identified 3,884 differentially expressed (DE) genes based on adjusted *P*-value ≤ 0.05 and investigated gene relationships based on weighted gene co-expression network analysis (WGCNA). After qRT-PCR validations, *MAP1LC3A*, *MAP1LC3B* (*P*-value < 0.01) and *BECN1* were found to show relatively higher expression levels in the treated groups than the control groups, while *PGC1α* (*P*-value < 0.05) showed the lower expressions. Accordingly, enrichment analyses of GO terms and KEGG pathways showed that several pathways [e.g., lysosome pathway (hsa04142) and p53 signaling pathway (hsa04115)] may be involved in the response of HK-2 cells to oxamate. Moreover, *via* WGCNA, we identified two modules: both the turquoise and blue modules were enriched in pathways associated with lysosome. However, the p53 signaling pathway was only found using all 3,884 DE genes. Furthermore, the key hub genes *IGFBP3* (adjusted *P*-value = 1.34×10^-75^ and log_2_(FC) = 2.64) interacted with 6 up-regulated and 12 down-regulated DE genes in the network that were enriched in the p53 signaling pathway. This is the first study reporting co-expression patterns of a gene network after lactate dehydrogenase inhibition in HK-2 cells. Our results may contribute to our understanding of the underlying molecular mechanism of *in vitro* reprogramming under hyperglycemic stress that orchestrates the survival and functions of HK-2 cells.

## Introduction

Diabetic kidney disease (DKD) is the leading cause of chronic kidney disease (CKD) and end-stage renal disease (ESRD) worldwide. Globally, due to the increasing incidence of diabetes and aging, DKD continues to increase stably and imposes the highest burden and the strongest correlation with mortality in patients with diabetes (1, 2). However, the pathological pathways that precipitate the development and/or progression of DKD remain to be fully elucidated. In the kidney, proximal tubular epithelial cells (PTECs), which represent ~90% of the outer kidney cortex and ~50% of the entire kidney mass, present more mitochondria than other renal cell types, because they reabsorb 80% of the filtrate that passes through the glomerulus, including large amounts of water, electrolytes, glucose, and other small molecules (3). Increased glucose reabsorption of PTECs under hyperglycemic stress triggers the initial period of hyperfiltration and tubule-glomerular feedback, exacerbates hypoxia and drives a poorly understood dominos of renal injuries including atrophy of proximal tubular, atubular glomeruli and tubulointerstitial fibrosis that associate with culminating in renal failure and further contributes to the emergence of proximal tubulocentric research (4, 5).

Recently, both *in vivo* and *in vitro* tracer studies have demonstrated an increased glycolysis-driven lactate production, or pyruvate-to-lactate production, in early renal changes associated with diabetes in rat kidney and PTECs (6). Lactic acid is the sink for three-carbon compounds, and the hub of glycolysis, mitochondrial energy metabolism and gluconeogenesis in PTECs (7). Pyruvate-to-lactate process reflects the redox buffer of NADH/NAD^+^ ratio across cells (7). Exploration of the pathophysiology involved mechanisms of lactate metabolic adaptation to reshape the field of metabolic reprogramming in hyperglycemic PTECs could offer a comprehensive update on strategies targeting renal tubules.

Oxamate is an isosteric and isoelectronic analogue of pyruvate, i.e., a lactate dehydrogenase (LDH) inhibitor wildly used in tumor cells or in tumor cell energy metabolism and apoptosis research. Recent studies found that oxamate could regulate glycemic homeostasis from both central and peripheral tissues (8). The previous results showed that oxamate, in rat proximal tubular cells line NRK-52E, impacted mitochondria respiration in a hyperglycemia-dependent manner (6). The flexibility of mitochondrial respiration adapting to nutrient stress is dependent on mitophagy and mitochondrial biogenesis; expressions of the mitophagy or mitochondrial biogenesis related genes and their encoded proteins might be involved, e.g., *BCL2*, *BECN1*, *MAP1LC3A*, *MAP1LC3B* and *PGC1α*. Therefore, we aim to explore the transcriptomics of human PTECs cultured with high D-glucose (HG) combined with oxamate *in vitro* to uncover the unexplored *in vitro* reprogramming under hyperglycemic stress and the underlying mechanism for this reprogramming that orchestrates the survival and functions of HK-2 cells (human cortex proximal tubular immortalized cell line), and the therapeutic potentials of targeting the pathways to reprogram the DKD.

The weighted gene co-expression network analysis (WGCNA) is widely used for gene co-expression networks that are constructed by genes with the significant co-expression relationships (9). It shows the co-expressed genes with the similar expression patterns across samples that are controlled by the same transcriptional regulatory programs (10, 11), which has been used in the integrated meta- and microRNA- analysis (12, 13); therefore it can be potentially used in the co-expression analysis of HK-2 cells after oxamate treatment. In this study, three independent replicates of human HK-2 cells with oxamate treatment and three independent replicates of control groups were used. The main objectives of our study are as follows:

1. Conduct a genome-wide transcriptome study on HK-2 cells in the absence and presence of oxamate treatment under HG condition to reveal differentially expressed (DE) genes.

2. Co-expressions analysis for HK-2 cells and construct the weighted gene co-expression networks.

3. Perform the enrichment analysis and identify the potential biological functions for the DE genes on HK-2 cells.

4. Reveal the candidate biomarkers to affect protein levels on HK-2 cells in the network.

## Materials and Methods

### HK-2 Cell Culture

HK-2 cells, the human renal proximal tubular epithelial cell line, were obtained from Beijing Beina Chuanglian Biotechnology Research Institute. HK-2 cells were cultured in Dulbeccos modified eagles medium (DMEM) with the low glucose (Sigma-Aldrich Inc, St. Louis, USA) supplemented with 10% fetal bovine serum (Sigma-Aldrich, St. Louis, USA), 10 units/ml penicillin and 10 mg/ml streptomycin. They were maintained in the continuous culture at 37°C in a humidified atmosphere (5% CO_2_) in an incubator. Growth medium was changed every second day, and cells were sub-cultured until further measurements at 80% colony confluency.

Oxamate could induce apoptosis in a dose-dependent manner (14); therefore, the xCELLigence real-time cell analyzer (RTCA) S16 system (Agilent Technologies, Santa Clara, USA) was used to monitor the dynamic real-time cell viability to explore a tolerable dose of oxamate. We exposed HK-2 cells to 5.5 mM low D-glucose (LG) medium or 25 mM HG medium with different concentrations of oxamate (i.e., 0 mM, 20 mM, 40 mM, 60 mM, 80 mM and 100 mM) for 24-hour intervention. One day prior cells were seeded at a density of 1×10^3^ cells per well, respectively, in the 16-well assay plate in medium containing 5.5 mM (LG) or 25 mM (HG) D-glucose. The medium was replaced by 5.5 mM LG medium or 25 mM HG medium with different concentrations of oxamate (i.e., 0 mM, 20 mM, 40 mM, 60 mM, 80 mM and 100 mM). Accordingly, we normalized the starting point 2 hours later after introducing the experimental variables and allowed the cells to grow for the oxamate intervening 24 hours *via* monitoring every 30 minutes. Here, each experiment was carried out in triplicate.

### Western Blot Analysis

The radioimmunoprecipitation assay lysis buffer (RIPA buffer) and phenylmethylsulfnoyl fluoride (PMSF) (Servicebio, Wuhan, China) were used to extract proteins at 4°C from the HK-2 cells treated with HG (25 mM) combined with or without different concentrations of oxamate (e.g., 0 mM, 20 mM, 40 mM, 80 mM) for 24 hours. We used BCA protein assay kit (Servicebio, Wuhan, China) to measure and adjust the protein concentrations. The proteins were denatured with 5 × SDS loading buffer (Servicebio, Wuhan, China) at 98°C for 15 min. Then, the prepared proteins were separated by SDS-polyacrylamide gel electrophoresis and concreted with 5% concentrated gel. Polyvinylidene fluoride membranes were used to transfer the proteins at 25 Voltage overnight. We used 5% skim milk solution to block the protein, incubated the membranes with the specific primary antibodies and the secondary antibodies, and visualized the membranes by ECL Plus reagents (Servicebio, Wuhan, China). The primary antibodies [Anti-PGC1α (ab54481), Anti-CASP3 (9665), Anti-BCL2 (ab59348), Anti-BAX (GB11690), Anti-BECN1 (bs-1353R), Anti-MAP1LC3 (14600-1-ap) and Anti-β-actin (GB12001)] and all secondary antibodies were purchased from abcom (Cambridge, MA, USA), Proteintech (Wuhan, China), BIOSS (Boston, Massachusetts, USA) and Servicebio (Wuhan, China), respectively. All primary antibodies were included at the ratio of 1: 3000. AlphaEaseFC software (Alpha Innotech, Miami, USA) was used to calculate the grayscale value of the proteins, and the protein images were processed by the Adobe software.

### RNA Sequencing, Read Quality Control and Alignment to Reference Genome

After HK-2 cell culture exposed to different concentrations of oxamate and western blot analysis, 40 mM was chosen as the appropriate oxamate treatment concentration. Thus, six replicates of cells were firstly exposed to HG treatments for seven days, then three replicates of them were exposed to 40 mM oxamate (case group), while the other three replicates were still under the same HG condition but 40 mM oxamate expose (control group). Unfortunately, one replicate of case group failed in RNA sample preparation, so five replicates of cells were finally chosen for RNA sequencing in this study. A total amount of 2 μg RNA per sample was used for RNA sample preparation. The sequencing libraries were generated using NEBNext Library Prep Kit (NEB, USA) for Illumina following the manufacturer’s recommendations. Then, they were sequenced on the Illumina Hiseq X ten platform to generate the 150 bp paired-end reads.

In order to guarantee the data quality, raw data was filtered for the clean reads by removing the contaminated reads for adapters (> 5 bp adapter sequences), low quality reads (Phred quality value <= 19 more than 15%) and reads with Ns (Ns > 5%). Afterwards, clean reads were aligned to the human reference genome GRCh38.p13 (Genome Reference Consortium Human Build 38) using HISAT2 software (version 2.1.0) (15) that uses a modified BWT algorithm to convert reference genome to index for faster speed and fewer resources.

### FPKM Calculation and Differentially Expressed Gene Analysis

We used HTSeq software (version 0.6.0) (16) to calculate the read counts for each gene. Then, the fragments per kilobase million mapped reads (FPKM) (**Supplementary File 1**) was calculated to estimate the expression levels of genes in each sample, following the formula


$$\text{FPKM}=\frac{10^{6}*F}{NL/10^{3}}$$

where F is the number of fragments in a certain sample that is assigned to a certain gene, N is the total number of mapped reads in the certain sample and L is the length of the certain gene. FPKM could eliminate the effect of sequencing depth and gene length on gene expression levels, so it was most commonly used in the previous studies (17, 18).

DE gene analysis between two groups was performed using R package DESeq2 (version 1.30.0) that was designed for differential gene expression analysis between two groups with biological replicate samples under the theoretical basis following the hypothesis of negative binomial distribution (19–21). DESeq2 estimates the expression level of each gene for each sample by the linear regression model to consider the genes of the same expression levels that share the similarity deviations or own the expression characteristics. It calculates the *P*-value by Wald test and corrects the multiple testings by Benjamini–Hochberg (BH) procedure to achieve the adjusted *P*-value. Here, genes with thresholds of adjusted *P*-value ≤ 0.05 are identified as DE genes. In addition, fold change (FC) of each gene was calculated based on the averaged FPKM value for case group and control group; thus, the up‐regulated and down‐regulated genes were defined when the log_2_(FC) values were positive and negative, respectively.

All genes were visualized in a volcano plot by the R function *plot* based on FCs and adjusted *P*-values. The DE genes were clustered in a heat map by the R function *heatmap* based on the transforms of log_10_(FPKM + 1) values, where the transforms were scaled for each gene and clustered using the default complete hierarchical clustering method.

### Differentially Expressed Gene Validation by qRT-PCR Experiment

The approach of qualitative reverse transcription polymerase chain reaction (qRT-PCR) was applied to validate five genes that are B-cell lymphoma 2 apoptosis regulator (*BCL2*), beclin1 (*BECN1*), microtubule-associated proteins 1A/1B light chain 3A (*MAP1LC3A*), microtubule-associated proteins 1A/1B light chain 3B (*MAP1LC3B*) and peroxisome proliferator-activated receptor-g coactivator 1α (*PGC1α*), using the re-cultured and re-treated cells. The selected genes might be involved in mitophagy and mitochondrial biogenesis for the flexibility of mitochondrial respiration adapting to nutrient stress. Glyceraldehyde-3-phosphate dehydrogenase (*GAPDH*) was chosen as the internal gene for qRT-PCR experiments and 2×SYBR Green qPCR Master Mix (High ROX) (Servicebio, Wuhan, China) was used to perform qRT-PCR experiments. The method of 2−^ΔΔCt^ was used to calculate the relative gene expression levels. All the primers for qRT-PCR experiments are shown in the **Table 1**.

**Table 1:** The primers used in the qRT-PCR experiments.

Gene name	Forward Primer	Reverse Primer	Length
*BCL2*	GAGGAAGTCCAATGTCCAGCC	GCATCCCAGCCTCCGTTATC	157
*BECN1*	ACATGAGCGAGTTGGTCAAGATC	CCCAGTGACCTTCAGTCTTCG	162
*MAP1LC3A*	ACATGAGCGAGTTGGTCAAGATC	ACATGAGCGAGTTGGTCAAGATC	141
*MAP1LC3B*	GTTGGCACAAACGCAGGGTA	ACACTGCTGCTTTCCGTAACAA	305
*PGC1α*	ACCCAGAACCATGCAAATCACA	ACCCAGAACCATGCAAATCACA	166
*GAPDH*	ACCCAGAACCATGCAAATCACA	ACCCAGAACCATGCAAATCACA	168

### Gene Co-Expression Network of Differentially Expressed Genes and Their Associations With Oxamate Treatment

The gene co-expression network was constructed by R package WGCNA (9) for the similarity measurement between the gene expression profiles by Pearson correlation coefficients of matrix. It transformed the similarity matrix into an adjacency matrix (A) raised to a β exponent (soft threshold) based on the free-scale topology model. A total of 20,377 genes were filtered out of 29,483 genes based on the median absolute deviation (MAD) ≥ 0.01 in this study. The β power parameter (soft threshold) was equal to 8 when the R^2^ of the free-scale topology was equal to 0.8 (**Supplementary Figure 1**).

In the network construction, we set the minimum module size equal to 30 for detection with the unsigned TOM type. Additionally, we set the dendrogram cut height for modules merging at 0.25 by clustering module eigengenes using the dissimilarity, so the modules whose eigengenes are highly correlated above 0.75 would be merged on each branch.

Module association between the module eigengenes (MEs) (i.e., the first principle component to represent the overall expression level of the module) and the oxamate treatment status (i.e., 0, 0, 0, 1, 1 for the control and case groups, respectively) was calculated for the relevant module identifications. We calculated the module significance (MS) to evaluate the correlations. Normally, modules with the highest MS score are considered as the key modules (9), thus MS genes in the association analysis (*P*-value < 0.1) were assigned for functional enrichment analysis.

### Gene Ontology and Pathway Enrichment Analysis

Both Gene Ontology (GO) terms and Kyoto Encyclopedia of Genes and Genomes (KEGG) pathways analysis were conducted to investigate the important enrichments for the associations between the identified DE genes and gene-related biological functions. We used R package clusterProfiler (version 3.6) (22) to test the statistical enrichments of GO terms and KEGG pathways using DE genes (adjusted *P*-value < 0.05) and DE genes in the key modules with high MS scores, where the GO and pathway enrichments were both based on over-representation analysis (ORA).

### Protein-Protein Interactive Analysis of Differentially Expressed Genes

In this study, protein-protein interactions (PPIs) were predicted for the top DE genes based on the STRING database (https://string-db.org). Blastx software (version 2.2.28) (https://blast.ncbi.nlm.nih.gov/Blast.cgi) was used to align the target gene sequences to the selected reference protein sequences using the default settings and the protein networks were built according to the known interactions of human species. Afterwards, we used Cytoscape software (version 3.5.1) to visualize the networks of PPIs (23).

## Results

### HK-2 Cell Culture and Tolerable Dose Exploration

**Figure 1:**
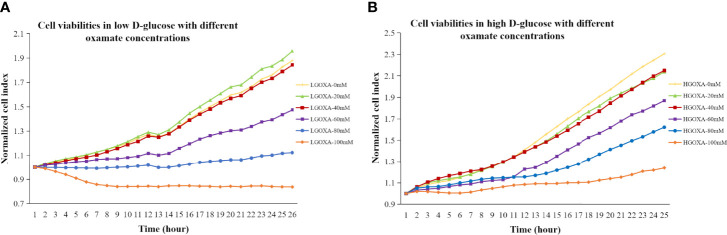
**(A)** Cell viabilities in low D-glucose (LG) with different concentrations of oxamate (LGOXA-0mM, LGOXA-20mM, LGOXA-40mM, LGOXA-60mM, LGOXA-80mM and LGOXA-100mM) for 24 hours. **(B)** Cell viabilities in high D-glucose (HG) with different concentrations of oxamate (HGOXA-0mM, HGOXA-20mM, HGOXA-40mM, HGOXA-60mM, HGOXA-80mM and HGOXA-100mM) for 24 hours.

Comparing with the cells cultured without oxamate (0 mM concentration), the cells treated with 20 mM or 40 mM oxamate retained the proliferation and viability either under LG or HG conditions. However, the cell viability progressively decreased after treating with even higher concentrations of oxamate (i.e., 60 mM, 80mM and 100mM) for 24 hours in both LG medium (**Figure 1A**) and HG medium (**Figure 1B**). It was suggested that oxamate affected the cell viability in a dose-dependent manner in either LG or HG culture condition within 24 hours (**Figure 1**).

**Figure 2:**
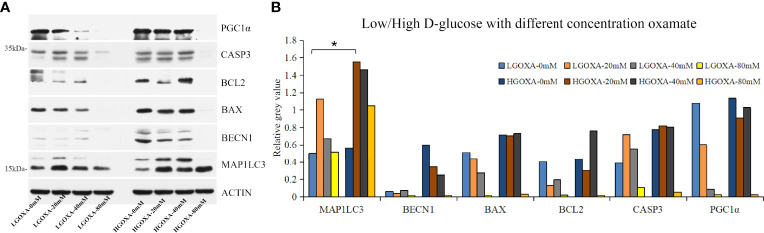
The protein expression levels of peroxisome proliferator-activated receptor-g coactivator 1α (PGC1α), caspase 3 (CASP3), B-cell lymphoma 2 apoptosis regulator (BCL2), BCL2 associated X apoptosis regulator (BAX), beclin1 (BECN1) and microtubule-associated proteins 1A/1B light chain 3 (MAP1LC3) in HK-2 cells treated with low D-glucose (LG) with different concentrations of oxamate (LGOXA-0mM, LGOXA-20mM, LGOXA-40mM and LGOXA-80mM) and high D-glucose **(HG)** with different concentrations of oxamate (HGOXA-0mM, HGOXA-20mM, HGOXA-40mM and HGOXA-80mM) for 24 hours. **(A)** Western blot gels. **(B)** Relative grey values. * indicates *P*-value < 0.05 after student’s *t*-test.

Further western blot assay was used to detect the protein expressions of PGC1α, CASP3, BCL2, BAX, BECN1 and MAP1LC3 (**Supplementary Figure 2**). The results showed that oxamate significantly decreased the protein expression levels of PGC1α and BAX under the culture of LGOXA-40mM comparing with the cells cultured without oxamate (0 mM concentration), whereas their expressions remained stable under the treatment of HGOXA-40mM (**Figures 2A, B**). Culturing HK-2 cells in 25 mM HG medium with 40 mM oxamate for 24 hours is suggested to prompt a diabetic state affiliated metabolic reprogramming without disrupting cell growth ability.

### Statistics of Alignment to Human Reference Genome

On average, 46,017,884 (97.14%) clean reads were retained from 47,372,938 raw reads after quality control by removing 251,576 low-quality reads, 110,726 ploy-N reads and 992,752 adapter polluted reads. Afterwards, 44,710,680 of them were uniquely mapped to the human reference genome with mapping rate of 97.16% (**Table 2**).

**Table 2:** Statistics of quality control and alignment to human reference genome (GRCh38.p13).

Sample	Raw read number	Low-quality read number (%)	Ploy-N read number (%)	Adapter polluted read number (%)	Clean read number (%)	Mapped read (%)
HG1	48,909,654	219,180 (0.45)	70,820 (0.14)	732,722 (1.50)	47,886,932 (97.91)	46,597,334 (97.31)
HG2	47,359,384	337,124 (0.71)	83,178 (0.18)	675,864 (1.43)	46,263,218 (97.69)	44,866,400 (96.98)
HG3	47,542,838	192,338 (0.41)	261,400 (0.55)	2,126,872 (4.47)	44,962,228 (94.57)	43,678,133 (97.14)
HGOXA2	46,419,566	243,330 (0.52)	60,204 (0.13)	698,242 (1.50)	45,417,790 (97.84)	44,197,980 (97.31)
HGOXA3	46,633,246	265,908 (0.57)	78,028 (0.17)	730,058 (1.57)	45,559,252 (97.70)	44,213,554 (97.05)
Mean	47,372,938	251,576 (0.53)	110,726 (0.23)	992,752 (2.09)	46,017,884 (97.14)	44,710,680 (97.16)
SD	980,639	55,115 (0.12)	84,669 (0.18)	634,430 (1.33)	1,144,377 (1.44)	1,135,766 (0.15)

% indicates the percentage. HGOXA2 and HGOXA3 indicate the case group that were treated with 40 mM oxamate in 25 mM HG medium. HG1, HG2 and HG3 indicate the control groups that were only treated with 25 mM HG medium without oxamate.

### Differentially Expressed Gene in Up/Down Regulation Status and Their Experimental Validations

In this study, we identified 3,884 DE genes (adjusted *P*-value ≤ 0.05) including 1,664 up-regulated DE genes and 2,220 down-regulated DE genes. All the details of 3,884 DE genes with gene names, read counts for each gene along different samples, log_2_(FC) values, *P*-values, adjusted *P*-values and regulation status are listed in **Supplementary File 2**. The top ten up-regulated DE genes and down-regulated DE genes were shown in **Table 3**. We found that the most up-regulated DE gene was *SAT1* (adjusted *P*-value = 5.57×10^-83^ and log_2_(FC) = 1.75), while the most down-regulated DE gene was *DYNC1H1* (adjusted *P*-value = 3.66×10^-41^ and log_2_(FC) = -1.14). In addition, the log_2_(FC) values varied from -1.68 to 3.16 among the top ten up-regulated DE genes and ten down-regulated DE genes (**Table 3**).

**Table 3:** Top ten up-regulated and ten down-regulated differentially expressed genes.

Ensembl gene ID	*Gene name*	Full description	Log_2_(FC)	Adjusted *P*-value	Regulation status
ENSG00000130066	*SAT1*	Spermidine/spermine N1-acetyltransferase 1	1.75	5.57×10^-83^	Up-regulated
ENSG00000146674	*IGFBP3*	Insulin like growth factor binding protein 3	2.64	1.34×10^-75^	Up-regulated
ENSG00000165272	*AQP3*	Aquaporin 3 (Gill blood group)	1.97	1.40×10^-59^	Up-regulated
ENSG00000126709	*IFI6*	Interferon alpha inducible protein 6	2.04	1.15×10^-53^	Up-regulated
ENSG00000163220	*S100A9*	S100 calcium binding protein A9	3.16	5.50×10^-51^	Up-regulated
ENSG00000187134	*AKR1C1*	Aldo-keto reductase family 1 member C1	2.12	8.45×10^-50^	Up-regulated
ENSG00000019186	*CYP24A1*	Cytochrome P450 family 24 subfamily A member 1	1.57	5.53×10^-49^	Up-regulated
ENSG00000169715	*MT1E*	Metallothionein 1E	1.42	1.07×10^-48^	Up-regulated
ENSG00000198886	*MT-ND4*	Mitochondrially encoded NADH dehydrogenase 4	1.04	2.15×10^-44^	Up-regulated
ENSG00000151632	*AKR1C2*	Aldo-keto reductase family 1 member C2	2.82	9.21×10^-43^	Up-regulated
ENSG00000197102	*DYNC1H1*	Dynein cytoplasmic 1 heavy chain 1	-1.14	3.66×10^-41^	Down-regulated
ENSG00000167548	*KMT2D*	Lysine methyltransferase 2D	-1.68	9.12×10^-38^	Down-regulated
ENSG00000109971	*HSPA8*	Heat shock protein family A (Hsp70) member 8	-1.03	1.58×10^-37^	Down-regulated
ENSG00000148773	*MKI67*	Marker of proliferation Ki-67	-1.38	6.53×10^-37^	Down-regulated
ENSG00000243156	*MICAL3*	Microtubule associated monooxygenase, calponin and LIM domain containing 3	-1.39	1.46×10^-35^	Down-regulated
ENSG00000149503	*INCENP*	Inner centromere protein	-1.36	5.14×10^-33^	Down-regulated
ENSG00000178209	*PLEC*	Plectin	-0.92	6.87×10^-33^	Down-regulated
ENSG00000127481	*UBR4*	Ubiquitin protein ligase E3 component n-recognin 4	-1.10	1.11×10^-30^	Down-regulated
ENSG00000113810	*SMC4*	Structural maintenance of chromosomes 4	-1.43	3.91×10^-29^	Down-regulated
ENSG00000130723	*PRRC2B*	Proline rich coiled-coil 2B	-1.10	1.44×10^-27^	Down-regulated

**Figure 3:**
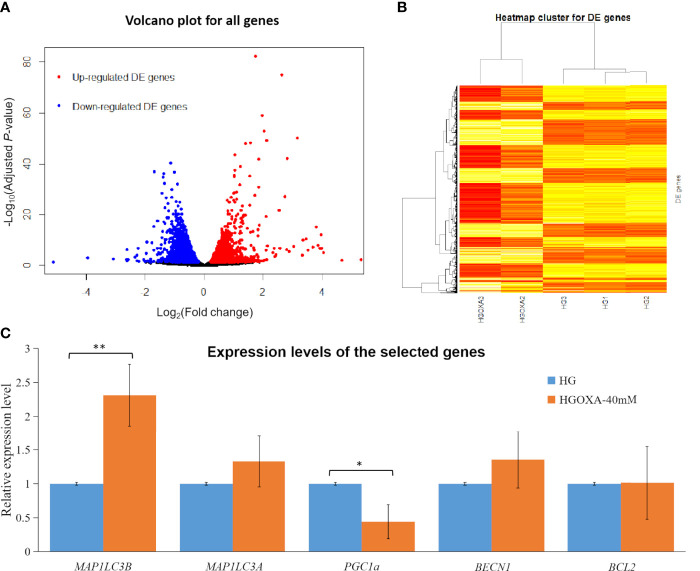
Differentially expressed (DE) analysis results. **(A)** Volcano plots for all genes based on fold change (FC) values and adjusted *P*-values. Note: Genes with thresholds of adjusted *P*-value ≤ 0.05 are identified as DE genes. **(B)** Heatmap plots for all DE genes. Note: The color from yellow to red indicates the gene expression levels from low to high after the transforms of log_10_(FPKM + 1) values. HGOXA2 and HGOXA3 indicate the case group that were treated with 40 mM oxamate in 25 mM HG medium. HG1, HG2 and HG3 indicate the control groups that were only treated with 25 mM HG medium without oxamate. **(C)** Relative expression levels of the selected genes validated by qRT-PCR experiments. Note: HGOXA-40mM indicates the case group that was treated with 40 mM oxamate in 25 mM HG medium. ** and * indicate the *P*-value < 0.01 and *P*-value < 0.05, respectively, after the student’s *t*-test.

**Figure 3** displayed the obvious division between up-regulated DE genes and down-regulated DE genes based on log_2_(FC) values. The range of log_2_(FC) values reached to -5 for down-regulated DE genes and 5 for up-regulated DE genes (**Figure 3A**). After the clustering of transformed FPKM values, we found that the DE genes with high expression levels (red color) in two samples (HGOXA2 and HGOXA3) from case group were clustered together, and vice versa (low expression levels in yellow color); thus, the gene expression levels showed an apparent partition between two treatment groups (**Figure 3B**). After the qRT-PCR experiment validations, we found that *MAP1LC3A*, *MAP1LC3B* (*P*-value < 0.01) and *BECN1* had relatively higher expression levels in the 40 mM oxamate treated groups than the control groups, while *PGC1α* (*P*-value < 0.05) showed the lower expressions (**Figure 3C**).

### Co-Expression Network of Differentially Expressed Genes and the Key Modules Associated With Oxamate Treatment

**Figure 4:**
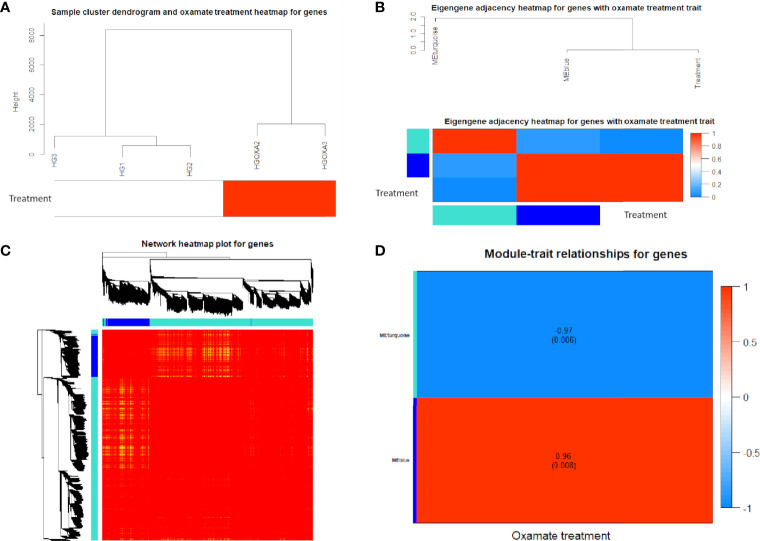
Co-expression network results of differentially expressed (DE) genes. **(A)** Sample cluster heatmap with oxamate treatment for DE genes. **(B)** Eigengene dendrogram and adjacency heatmap of different co-expression modules for DE genes. **(C)** Weighted DE gene network heatmap of the co-expression interactions based on the topological overlap matrix (TOM) dissimilarity. Note: The gene dendrogram and module assignment are shown along the left side and the top, where the axe colors indicate the different modules. The color intensity inside the heatmap represents the overlap degree, where light color represents low overlap and darker red color represents higher overlap. **(D)** Module-trait relationship heatmap between oxamate treatment and control groups for DE genes. Each row indicates module eigengenes (the first principle component of the module) with the correlation coefficients (*P*-values in the brackets), where red color represents positive correlation and blue color represents negative correlation.

Using 3,884 DE genes for the sample clustering, we found the samples with oxamate treatment were clustered together (**Figure 4A**). The eigengene adjacency heatmap indicated that these modules of DE genes could be clustered further together into groups, where treatment status was grouped with blue pattern (**Figure 4B**). The weighted DE gene network construction was visualized in the topological overlap matrix (TOM) clusters that showed a high level of overlap densities among the two clusters (**Figure 4C**). Obviously, DE genes were grouped into 2 modules that had similar co-expressions using the average linkage hierarchical clustering algorithm (**Figures 4B, C**), where 3,087 DE genes were grouped into turquoise module as the key module, followed by 797 DE genes into blue module. The module-trait relationship results revealed that oxamate treatment had high correlations with the turquoise module (correlation coefficient = -0.97, *P*-value < 0.01) and blue module (correlation coefficient = 0.96, *P*-value < 0.01) (**Figure 4D**).

### Significant Enrichments of GO Term and Pathway

**Figure 5:**
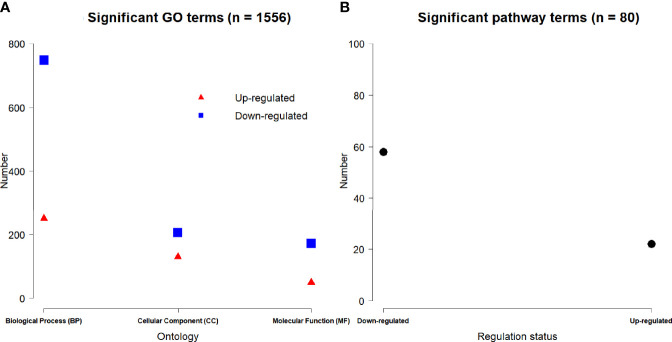
Number of the significant up-regulated and down-regulated **(A)** GO terms and **(B)** pathways.

Based on 3,884 DE genes, a total of 1,556 significant GO terms were derived for three domains including 998 biological process (BP), 336 cellular component (CC) and 222 molecular function (MF) ontologies (**Figure 5A**). Based on the regulation status of DE genes, 430 up-regulated and 1126 down-regulated significant GO terms (**Figure 5A**), and 22 up-regulated and 58 down-regulated significant pathways (**Figure 5B**) were achieved respectively.

**Figure 6:**
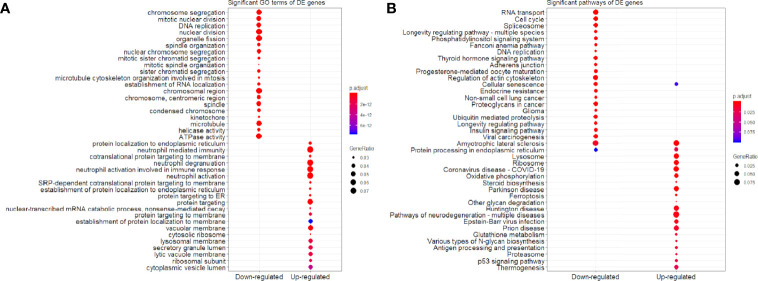
Significant **(A)** GO terms and **(B)** pathways for differentially expressed (DE) genes (n = 3,884) in the down-regulated and up-regulated categories.

**Figure 7:**
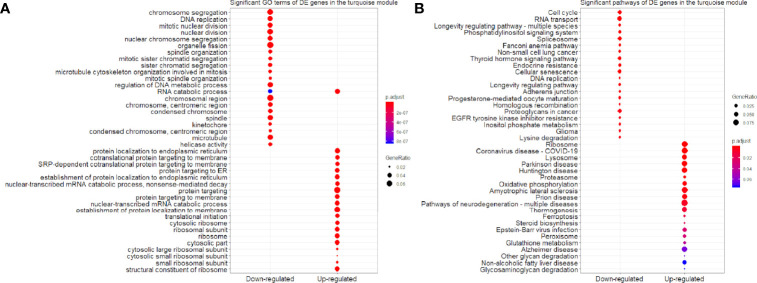
Significant **(A)** GO terms and **(B)** pathways for differentially expressed (DE) genes in the turquoise module (n = 3,087) in the down-regulated and up-regulated categories.

**Figure 8:**
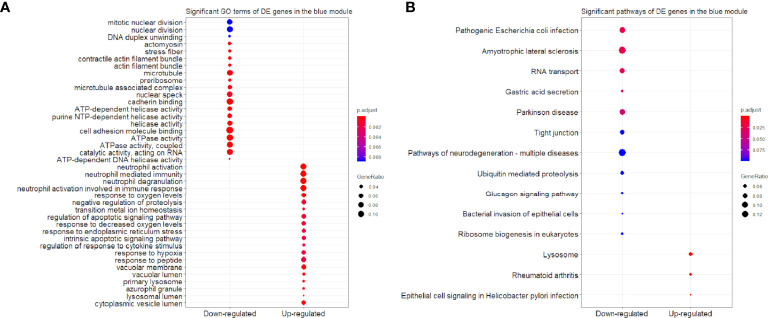
Significant **(A)** GO terms and **(B)** pathways for differentially expressed (DE) genes in the blue module (n = 797) in the down-regulated and up-regulated categories.

We found that the three most significant GO terms were chromosome segregation (GO:0007059, adjusted *P*-value = 2.95×10^-30^) with 115 DE genes, mitotic nuclear division (GO:0140014, adjusted *P*-value = 2.33×10^-28^) with 104 DE genes and DNA replication (GO:0006260, adjusted *P*-value = 2.33×10^-28^) with 102 DE genes in the BP ontology of down-regulated category (**Figure 6A**). Similarly, the three most significant pathways were also in the down-regulated category including RNA transport (hsa03013, adjusted *P*-value = 1.43×10^-9^) with 55 DE genes, cell cycle (hsa04110, adjusted *P*-value = 2.04×10^-9^) with 42 DE genes and spliceosome (hsa03040, adjusted *P*-value = 1.27×10^-6^) with 42 DE genes (**Figure 6B**). Based on 3,087 DE genes in the turquoise module after WGCNA analysis, the three most significant GO terms and pathways (**Figures 7A, B**) were similar to the enrichment results based on 3,884 DE genes, except the longevity regulating pathway - multiple species (hsa04213, adjusted *P*-value = 3.46×10^-6^) with 22 DE genes (**Figure 7B**) only in the turquoise module. However, enrichment results of 797 DE genes in the blue module were quite different from those in the turquoise module (**Figures 8A, B**), where only RNA transport was still one of the three most significant pathways (**Figure 8B**).

In the up-regulated category, protein localization to endoplasmic reticulum (GO:0070972, adjusted *P*-value = 6.26×10^-18^, DE gene = 51), neutrophil mediated immunity (GO:0002446, adjusted *P*-value = 1.14×10^-17^, DE gene = 102) and cotranslational protein targeting to membrane (GO:0006613, adjusted *P*-value = 1.36×10^-17^, DE gene = 43) in the BP ontology were the most significant GO terms based on 3,884 DE genes (**Figure 6A**). Meanwhile, lysosome (hsa04142, adjusted *P*-value = 2.30×10^-13^, DE gene = 45), ribosome (hsa03010, adjusted *P*-value = 3.54×10^-11^, DE gene = 47) and coronavirus disease - COVID-19 (hsa05171, adjusted *P*-value = 5.07×10^-8^, DE gene = 53) were the most significant pathways (**Figure 6B**). Moreover, we found that p53 signaling pathway with 16 DE genes (hsa04115, adjusted *P*-value = 1.73×10^-2^) was also involved in the top significant pathways (**Figure 6B**). Based on 3,087 DE genes in the turquoise module, the three most significant GO terms and pathways (**Figures 7A, B**) were similar to the enrichment results based on 3,884 DE genes, but enrichment results of 797 DE genes in the blue module were still different (**Figures 8A, B**).

### Protein-Protein Interaction Networks of Differentially Expressed Genes

**Figure 9:**
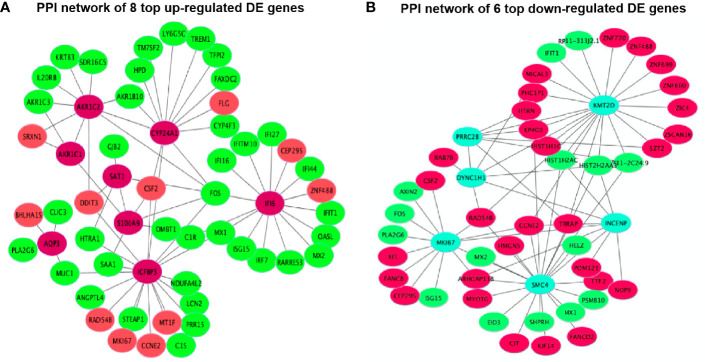
Protein-protein interactions (PPIs) network of 14 top DE genes including **(A)** 8 top up-regulated DE genes and **(B)** 6 top down-regulated DE genes.

The PPI network results were visualized using the top eight up-regulated DE genes (*SAT1*, *IGFBP3*, *AQP3*, *IFI6*, *S100A9*, *AKR1C1*, *CYP24A1* and *AKR1C2*) and the top six down-regulated DE genes (*DYNC1H1, KMT2D, INCENP, SMC4, MKI67* and *PRRC2B*). It showed that most DE genes had the PPIs with at least two other DE genes (**Figure 9**). For example, the up-regulated *IGFBP3* had the PPIs with the other 6 up-regulated DE genes (*CSF2*, *MT1F*, *RAD54B*, *MKI67*, *CCNE2* and *DDIT3*) and 12 down-regulated DE genes (*STEAP1*, *ANGPTL4*, *FOS*, *PRR15*, *HTRA1*, *LCN2*, *C1R*, *MUC1*, *SAA1*, *NDUFA4L2*, *MX1* and *C1S*) (**Figure 9A**). The down-regulated *KMT2D* had the PPIs with the other 13 up-regulated DE genes (*RP11-313J2.1*, *ZNF699*, *CCNE2*, *ZNF488*, *ZNF770*, *RAD548*, *SZT2*, *EP400*, *MICAL3*, *TRRAP*, *DYNC1H1*, *ZIC4* and *PHC1P1*) and 6 down-regulated DE genes (*HIST2H2AA3*, *ZSCAN16*, *HIST1H1C*, *ZNF880*, *HIST1H2AC* and *IFIT1*) (**Figure 9B**).

## Discussion

Proximal tubules present a very high density of mitochondria required for energy consumption (24). Mitochondria occupy about 16.3% relative volume of the human cross-sectioned proximal convoluted tubules (S1 and S2 combined) (25). Mitochondrial dysfunction, with persistent energy depletion and deficiency of oxygen utilization efficiency, is the main driver in the progression of hyperglycemic proximal tubules (24). The process of selective removal of dysfunctional and depolarized mitochondria, known as mitophagy, interfaces and coordinates with mitochondrial biogenesis to maintain mitochondrial homeostasis. Previous studies showed that oxamate could impact mitochondria respiration in a hyperglycemia-dependent manner in the rat proximal tubular cells (6). Here, our results further suggested that under diabetic state, this metabolic reprogramming caused by oxamate was related to its impact on the resilience in both mitophagy and mitochondrial biogenesis, in which MAP1LC3 (MAP1LC3A and MAP1LC3B), BECN1 and PGC1α were involved.

### Oxamate Features on Cell Viability/Survival and Mitochondrial Biogenesis

In mammals, lactate and pyruvate as two sinks for three-carbon compounds, enable the crosstalk between glycolytic flux and mitochondrial respiratory function, and serve as a circulating redox buffer that equilibrates the NADH/NAD+ ratio across cells and tissues together (7). For a long time, oxamate had been considered as a classic LDH enzyme inhibitor. Later, the kinetic modeling results revealed the multiple sites targeting of oxamate (14). Besides LDH inhibition function, oxamate strongly blocked pryruvate kinase (PYK) and enolase (ENO) activities, and slightly inhibited hexosephosphate isomerase (HPI), aldolase (ALD) and glucose-6-phosphate dehydrogenas (Glc6PDH) activities (14). Oxamate effect on cell fate in a dose-dependent manner depended on its cumulative influences on multiple combinations of metabolic enzymes and metabolites. Heretofore, we found that treating hyperglycemic NRK-52E with a relatively high concentration of oxamate (e.g., 100mM) significantly reduced the cell proliferations and survivals (6). To avoid the bias related to cell death caused by excessive drug concentration, we explored the dose-dependent manner of oxamate on cell viability/survival and mitochondrial biogenesis. Finally, cells treated with 40mM oxamate under HG condition that retained cell viability/survival is suggested to abrogate the PGC1α inhibiting properties caused by oxamate (**Figures 1**, **2**); thus, 40mM oxamate is considered as a plausible concentration for our transcriptomic study.

### Differentially Expressed and Co-Expressed Genes After Oxamate Treatment

Oxamate was found to downregulate the expression of *IL6* and upregulate the expression of *PDE3B* in the skeletal muscle of *db/db* mice (26). However, *IL6* (adjusted *P*-value = 2.43×10^-2^ and log_2_(FC) = 0.54) was identified as one up-regulated DE gene, whereas *PDE3B* (adjusted *P*-value = 1.80×10^-2^ and log_2_(FC) = -0.52) as one down-regulated DE gene in our study (**Supplementary File 2**). As a B-cell differentiation factor, interleukin 6 (IL6) is a multifunctional cytokine to regulate the hematopoiesis, immune and acute-phase responses, and inflammations of interleukin 1 (IL1), tumor necrosis factor alpha (TNF-α), and lipopolysaccharide (LPS) as the stimuli (27–30). *PDE3B* with *PDE3A* from the PDE3 family hydrolyze cAMP and cGMP, and its isoforms are in higher expressions than *PDE3A* in tissues that regulate energy homeostasis, including adipose tissue, liver and pancreatic β cells (31, 32). Ahmad et al. (33) demonstrated that the role of *PDE3B* regulated NLRP3 inflammasome in modulating inflammatory responses to contribute to a reduced inflammatory state in adipose tissue. In addition, our study found *SAT1* (Spermidine/spermine N1-acetyltransferase 1) was the most up-regulated DE gene (adjusted *P*-value = 5.57×10^-83^ and log_2_(FC) = 1.75) (**Table 3**). It is reported that *SAT1* is an important transporter involved in sulfate homeostasis as an anion exchanger and regulates the expression of the hepatocellular sulfate by glyoxylate that could be a metabolic link between liver and kidney (34).

The HIF inhibiting properties were removed using oxamate in the hyperglycemic rat proximal tubular cells (6). Studies have been reported that oxamate could induce autophagy *via* downregulation HIF-1α through inhibiting the Akt-mTOR signaling pathway in cancer cells (35, 36). *BECN1* is a haploinsufficient tumor suppressor gene as a core component of the class III phosphatidylinositol 3-kinase that is involved in autophagosome formation and vesicular trafficking (37). It has been suggested that *BECN1* was required for mitophagy completion, and suppressed mitophagy by *BECN1* deficiency could cause aberrant mitochondria quality control in adipocyte to cause lipodystrophy and metabolic dysregulation (38). The qRT-PCR experiment results of *MAP1LC3A* (adjusted *P*-value = 1.30×10^-3^ and log_2_(FC) = 0.71) and *MAP1LC3B* (adjusted *P*-value = 8.47×10^-4^ and log_2_(FC) = 0.35) showed higher expression levels in the 40 mM oxamate treated groups than the non-treated groups after validations (*P*-value < 0.01 after student’s *t*-test) (**Figure 3C**), which is consistent with the transcriptome results in the up-regulated status (**Supplementary File 2**). Matboli et al. (2017) (39) revealed *MAP1LC3A* with the high diagnostic power for detection of DKD, as the urinary expression level of *MAP1LC3A* was significantly lower in DKD than the control group. The mRNA expressions of *MAP1LC3B* were also down-regulated in the DKD group (40). MAP1LC3 is essential in autophagosome mature and selective engulfment of damaged mitochondria fusing with lysosomes and processing mitophagy (41, 42). Therefore, oxamate could be involved in reprogramming the DKD *via* the influence of some potential therapeutic pathways, where the DE *MAP1LC3A* and *MAP1LC3B* are involved.

### Significant Pathways and Protein-Protein Interaction Networks in Human Proximal Tubular Cells

Lysosome (hsa04142) was identified as one of the common significant pathways based on 3,884 DE genes, 3,087 DE genes in the turquoise module and 797 DE genes in the blue module (**Figures 6B**, **7B**, **8B**), where *ACP2*, *AGA*, *ARSA*, *ASAH1*, *ATP6AP1*, *CD63*, *CTNS*, *CTSA*, *CTSB, CTSC*, *CTSD*, *CTSF*, *CTSH*, *CTSL*, *CTSS*, *CTSZ*, *DNASE2*, *FUCA1*, *FUCA2*, *GAA*, *GLA*, *GNPTG*, *GUSB*, *HEXA*, *HEXB*, *HGSNAT*, *HYAL3*, *LAMP1*, *LAPTM4A*, *LAPTM4B*, *LGMN*, *LIPA*, *LITAF*, *MAN2B1*, *NAGLU*, *NAGPA*, *NAPSA*, *NEU1*, *NPC2*, *PPT1*, *PSAP*, *SLC11A2*, *SMPD1 *and *TPP1* were enriched in. Lysosome, known as the place for autophagy, has been recognized as a highly dynamic organelle that bidirectionally contact with mitochondria, to sense nutrient stress and control the switch between anabolism and catabolism by regulating mitochondrial biogenesis and autophagy (43, 44).

There is one pathway, p53 signaling pathway (hsa04115), only found in all 3,884 DE gene enrichment (**Figure 6B**), where *AIFM2*, *BBC3*, *BCL2L1*, *BID*, *CCNE1*, *CDKN1A*, *CHEK2*, *COP1*, *GADD45A*, *GADD45B*, *IGFBP3*, *PERP*, *SHISA5*, *SIAH1*, *TP53* and *TP53I3* were enriched. Chen et al. (45) revealed that MPC-5 cells from the high glucose-induced proliferation-inhibition and apoptosis-promotion *via* p53 signaling pathway can be protected by silencing the CCNG1 (45). Here, *IGFBP3*, as one of the key genes, interacted with 6 up-regulated and 12 down-regulated DE genes in the PPI networks (**Figure 9A**). It is reported that increased oxidative stress from high glucose enhances IGFBP3 expression to induce apoptosis; however, increased IGFBP3 expression by high glucose mediates high-glucose-induced apoptosis in PTECs and induces additional oxidative stress, which may result in amplification of hyperglycemic damage (46–48). The inhibition of LDH in hyperglycemic proximal tubular increased aerobic glycolysis (6), and changes in the extramitochondrial-free NADH/NAD+ ratio signal associated with aerobic glycolysis could control the abundance and activity of p53 by the C-terminal binding protein (CtBP) family of NADH-sensitive transcriptional regulators (49). Previous studies showed increased NADH/NAD+ ratio in response to the oxamate treatment in hyperglycemic rat proximal tubular cells (6). Here, we propose that the NADH-CtBP-p53 pathway may present as one of the hyperglycemia-dependent metabolic reprogramming caused by oxamate.

## Conclusions

In summary, our study conducted the genome-wide transcriptome and co-expression analysis for HK-2 cells to identify 3,884 DE genes (adjusted *P*-value ≤ 0.05), where 1,664 of them were up-regulated and 2,220 of them were down-regulated. In addition, two modules of co-expression patterns were constructed to perform GO terms and pathways. We found that lysosome (hsa04142) can be enriched in DE genes of all, turquoise and blue modules, but p53 signaling pathway (hsa04115) was only detected in all DE genes. *IGFBP3* (adjusted *P*-value = 1.34×10^-75^ and log_2_(FC) = 2.64), one of the key genes in p53 signaling pathway (hsa04115), interacted with several DE genes in the PPI networks and could be considered as the candidate biomarker due to its impact in high glucose conditions, after further validations. Together, our results highlight a possibility that anaerobic glycolysis-induced lactate in hyperglycemic HK-2 cells might integrate metabolic signals to mitochondria-lysosome contacts through some unexplored mechanism, which oscillates the coupling of mitochondrial biogenesis and mitophagy, and prolongedly compromises stress resistance.

## Data Availability Statement

The raw RNA sequencing data were deposited in the Gene Expression Omnibus (GEO) of National Center for Biotechnology Information (NCBI) with the accession number GSE182138 at https://www.ncbi.nlm.nih.gov/geo/query/acc.cgi?acc=GSE182138.

## Author Contributions

ZW, XW, and GQ conceived and designed the experiments. ZW and JY performed the experiments. XW, DH, and ZW analyzed the data and wrote the manuscript. ZW, DH, DF, XW, and GQ improved the manuscript. All authors read and approved the final manuscript.

## Funding

This study was supported by the Overseas Research Project of Scientific and Technological Talents in Health and Family Planning of Henan Province (No. 2015013) and National Natural Science Foundation of China (No. 82100896).

## Conflict of Interest

XW was employed by company Konge Larsen ApS.

The remaining authors declare that the research was conducted in the absence of any commercial or financial relationships that could be construed as a potential conflict of interest.

## Publisher’s Note

All claims expressed in this article are solely those of the authors and do not necessarily represent those of their affiliated organizations, or those of the publisher, the editors and the reviewers. Any product that may be evaluated in this article, or claim that may be made by its manufacturer, is not guaranteed or endorsed by the publisher.

## Supplementary Material

Supplementary Figure 1 R^2^ of the free-scale topology and mean connectivity with soft threshold (power) for 20,377 genes.

Supplementary Figure 2 The original Western blot gels of protein levels of peroxisome proliferator-activated receptor-g coactivator 1α (PGC1α), caspase 3 (CASP3), B-cell lymphoma 2 apoptosis regulator (BCL2), BCL2 associated X apoptosis regulator (BAX), beclin1 (BECN1) and microtubule-associated proteins 1A/1B light chain 3 (MAP1LC3) in HK-2 cells treated with low D-glucose (LG) with different concentrations of oxamate (LGOXA-0mM, LGOXA-20mM, LGOXA-40mM and LGOXA-80mM) and high D-glucose (HG) with different concentrations of oxamate (HGOXA-0mM, HGOXA-20mM, HGOXA-40mM and HGOXA-80mM) for 24 hours.

Supplementary File 1 The fragments per kilobase million mapped reads (FPKM) for each gene (n = 29,483) along five different samples.

Supplementary File 2 All 3,884 differentially expressed (DE) genes with gene names, read counts, log_2_(FC) values, *P*-values, adjusted *P*-values and regulation status along five different samples.

## References

[1] Anders HJ Huber TB Isermann B Schiffer M. CKD in Diabetes: Diabetic Kidney Disease Versus Nondiabetic Kidney Disease. Nat Rev Nephrol (2018) 14:361–77. doi: 10.1038/s41581-018-0001-y 29654297

[2] Bonner R Albajrami O Hudspeth J Upadhyay A. Diabetic Kidney Disease. Primary Care - Clinics Office Pract (2020) 47:645–59. doi: 10.1016/j.pop.2020.08.004 33121634

[3] Bhargava P Schnellmann RG. Mitochondrial Energetics in the Kidney. Nat Rev Nephrol (2017) 13:629–46. doi: 10.1038/nrneph.2017.107 PMC5965678 28804120

[4] Vallon V Thomson SC. The Tubular Hypothesis of Nephron Filtration and Diabetic Kidney Disease. Nat Rev Nephrol (2020) 16:317–36. doi: 10.1038/s41581-020-0256-y PMC7242158 32152499

[5] Duan S Lu F Song D Zhang C Zhang B Xing C. Current Challenges and Future Perspectives of Renal Tubular Dysfunction in Diabetic Kidney Disease. Front Endocrinol (2021) 12:661185. doi: 10.3389/fendo.2021.661185 PMC8223745 34177803

[6] Wang Z Nielsen PM Laustsen C Bertelsen LB. Metabolic Consequences of Lactate Dehydrogenase Inhibition by Oxamate in Hyperglycemic Proximal Tubular Cells. Exp Cell Res (2019) 378:51–6. doi: 10.1016/j.yexcr.2019.03.001 30836064

[7] Rabinowitz JD Enerbäck S. Lactate: The Ugly Duckling of Energy Metabolism. Nat Metab (2020) 2:566–71. doi: 10.1038/s42255-020-0243-4 PMC7983055 32694798

[8] Lam TK Gutierrez-Juarez R Pocai A Rossetti L. Regulation of Blood Glucose by Hypothalamic Pyruvate Metabolism. Science (2005) 309(5736):943–7. 10.1126/science.1112085 16081739

[9] Langfelder P Horvath S. WGCNA: An R Package for Weighted Correlation Network Analysis. BMC Bioinf (2008) 9:559. doi: 10.1186/1471-2105-9-559 PMC2631488 19114008

[10] Stuart JM Segal E Koller D Kim SK. A Gene-Coexpression Network for Global Discovery of Conserved Genetic Modules. Science (2003) 302(5643):249–255. doi: 10.1126/science.1087447 12934013

[11] Weirauch MT. Gene Coexpression Networks for the Analysis of DNA Microarray Data. Appl Stat Netw Biol: Methods Syst Biol (2011) 1:215–50. doi: 10.1002/9783527638079.ch11

[12] Farhadian M Rafat SA Panahi B Mayack C. Weighted Gene Co-Expression Network Analysis Identifies Modules and Functionally Enriched Pathways in the Lactation Process. Sci Rep (2021) 11:1–15. doi: 10.1038/s41598-021-81888-z 33504890 PMC7840764

[13] Hao D Wang X Yang Y Thomsen B Holm L-E Qu K. Integrated Analysis of mRNA and MicroRNA Co-Expressed Network for the Differentiation of Bovine Skeletal Muscle Cells After Polyphenol Resveratrol Treatment. Front Vet Sci (2021) 8:777477. doi: 10.3389/fvets.2021.777477 35036414 PMC8759604

[14] Moreno-Sánchez R Marín-Hernández Á. Del Mazo-Monsalvo I Saavedra E Rodríguez-Enríquez S. Assessment of the Low Inhibitory Specificity of Oxamate, Aminooxyacetate and Dichloroacetate on Cancer Energy Metabolism. Biochim Biophys Acta - Gen Subj (2017) 1861:3221–36. doi: 10.1016/j.bbagen.2016.08.006 27538376

[15] Kim D Langmead B Salzberg SL. HISAT: A Fast Spliced Aligner With Low Memory Requirements. Nat Methods (2015) 12:357–60. doi: 10.1038/nmeth.3317 PMC4655817 25751142

[16] Anders S Pyl PT Huber W. HTSeq-A Python Framework to Work With High-Throughput Sequencing Data. Bioinformatics (2015) 31:166–9. doi: 10.1093/bioinformatics/btu638 PMC4287950 25260700

[17] Trapnell C Williams BA Pertea G Mortazavi A Kwan G Van Baren MJ. Transcript Assembly and Quantification by RNA-Seq Reveals Unannotated Transcripts and Isoform Switching During Cell Differentiation. Nat Biotechnol (2010) 28:511–5. doi: 10.1038/nbt.1621 PMC3146043 20436464

[18] Dan Hao Wang X Wang X Thomsen B N.Kadarmideen H Lan X. Transcriptomic Changes in Bovine Skeletal Muscle Cells After Resveratrol Treatment. Gene (2020) 754:144849. doi: 10.1016/j.gene.2020.144849 32512157

[19] Wang L Feng Z Wang X Wang X Zhang X. DEGseq: An R Package for Identifying Differentially Expressed Genes From RNA-Seq Data. Bioinformatics (2009) 26:136–8. doi: 10.1093/bioinformatics/btp612 19855105

[20] Anders S Huber W. Differential Expression Analysis for Sequence Count Data. Genome Biol (2010) 11:R106. doi: 10.1186/gb-2010-11-10-r106 20979621 PMC3218662

[21] Love MI Huber W Anders S. Moderated Estimation of Fold Change and Dispersion for RNA-Seq Data With Deseq2. Genome Biol (2014) 15:550. doi: 10.1186/s13059-014-0550-8 25516281 PMC4302049

[22] Yu G Wang LG Han Y He QY. Clusterprofiler: An R Package for Comparing Biological Themes Among Gene Clusters. Omics A J Integr Biol (2012) 16:284–7. doi: 10.1089/omi.2011.0118 PMC3339379 22455463

[23] Shannon P Markiel A Ozier O Baliga NS Wang JT Ramage D. Cytoscape: A Software Environment for Integrated Models of Biomolecular Interaction Networks. Genome Res (2003) 13:2498–504. doi: 10.1101/gr.1239303 PMC403769 14597658

[24] Forbes JM Thorburn DR. Mitochondrial Dysfunction in Diabetic Kidney Disease. Nat Rev Nephrol (2018) 14:291–312. doi: 10.1038/nrneph.2018.9 29456246

[25] Møller JC Skriver E. Quantitative Ultrastructure of Human Proximal Tubules and Cortical Interstitium in Chronic Renal Disease (Hydronephrosis). Virchows Archiv A Pathological Anat Histopathol (1985) 406:389–406. doi: 10.1007/BF00710231 3925616

[26] Ye W Zheng Y Zhang S Yan L Cheng H Wu M. Oxamate Improves Glycemic Control and Insulin Sensitivity *via* Inhibition of Tissue Lactate Production in Db/Db Mice. PloS One (2016) 11:1–19. doi: 10.1371/journal.pone.0150303 PMC4777529 26938239

[27] Boswell RN Yard BA Schrama E Van Es LA Daha MR van der Woude FJ. Interleukin 6 Production by Human Proximal Tubular Epithelial Cells *In Vitro*: Analysis of the Effects of Interleukin-1α (IL-1α) and Other Cytokines. Nephrol Dialysis Transplant (1994) 9:599–606. doi: 10.1093/ndt/9.6.599 7970084

[28] Hirano T. Interleukin 6 and Its Receptor: Ten Years Later. Int Rev Immunol (1998) 16:249–84. doi: 10.3109/08830189809042997 9505191

[29] Leonard M Ryan MP Watson AJ Schramek H Healy E. Role of MAP Kinase Pathways in Mediating IL-6 Production in Human Primary Mesangial and Proximal Tubular Cells. in. Kidney Int (1999) 56(4):1366–77. doi: 10.1046/j.1523-1755.1999.00664.x 10504489

[30] Kamimura D Hirano T Murakami M. Interleukin-6. In: The Curated Reference Collection in Neuroscience and Biobehavioral Psychology. Elsevier Science Ltd., (2016). pp. 430–9.

[31] Degerman E Ahmad F Chung YW Guirguis E Omar B Stenson L. From PDE3B to the Regulation of Energy Homeostasis. Curr Opin Pharmacol (2011) 11:676–82. doi: 10.1016/j.coph.2011.09.015 PMC3225700 22001403

[32] Maurice DH Ke H Ahmad F Wang Y Chung J Manganiello VC. Advances in Targeting Cyclic Nucleotide Phosphodiesterases. Nat Rev Drug Discov (2014) 13:290–314. doi: 10.1038/nrd4228 24687066 PMC4155750

[33] Ahmad F Chung YW Tang Y Hockman SC Liu S Khan Y. Phosphodiesterase 3b (PDE3B) Regulates NLRP3 Inflammasome in Adipose Tissue. Sci Rep (2016) 6:28056. doi: 10.1038/srep28056 27321128 PMC4913246

[34] Stieger B. Regulation of the Expression of the Hepatocellular Sulfate-Oxalate Exchanger SAT-1 (SLC26A1) by Glyoxylate: A Metabolic Link Between Liver and Kidney? J Hepatol (2011) 54:406–7. doi: 10.1016/j.jhep.2010.09.011 21084130

[35] Zhao Z Han F Yang S Wu J Zhan W. Oxamate-Mediated Inhibition of Lactate Dehydrogenase Induces Protective Autophagy in Gastric Cancer Cells: Involvement of the Akt-mTOR Signaling Pathway. Cancer Lett (2015) 358:17–26. doi: 10.1016/j.canlet.2014.11.046 25524555

[36] Coronel-Hernández J Salgado-García R Cantú-De León D Jacobo-Herrera N Millan-Catalan O Delgado-Waldo I. Combination of Metformin, Sodium Oxamate and Doxorubicin Induces Apoptosis and Autophagy in Colorectal Cancer Cells *via* Downregulation HIF-1α. Front Oncol (2021) 11:594200. doi: 10.3389/fonc.2021.594200 34123772 PMC8187873

[37] Wirawan E Lippens S Berghe TV Romagnoli A Fimia GM Piacentini M. Beclin 1: A Role in Membrane Dynamics and Beyond. Autophagy (2012) 8:6–17. doi: 10.4161/auto.8.1.16645 22170155

[38] Jin Y Ji Y Song Y Choe SS Jeon YG Na H. Depletion of Adipocyte Becn1 Leads to Lipodystrophy and Metabolic Dysregulation. Diabetes (2021) 70:182–95. doi: 10.2337/db19-1239 PMC7881852 33046512

[39] Matboli M Azazy AEM Adel S Bekhet MM Eissa S. Evaluation of Urinary Autophagy Transcripts Expression in Diabetic Kidney Disease. J Diabetes Complications (2017) 31:1491–8. doi: 10.1016/j.jdiacomp.2017.06.009 28760651

[40] Matboli M Eissa S Ibrahim D Hegazy MGA Imam SS Habib EK. Caffeic Acid Attenuates Diabetic Kidney Disease via Modulation of Autophagy in a High-Fat Diet/Streptozotocin- Induced Diabetic Rat. Sci Rep (2017) 7:2263. doi: 10.1038/s41598-017-02320-z 28536471 PMC5442114

[41] Nguyen TN Padman BS Usher J Oorschot V Ramm G Lazarou M. Atg8 Family LC3/GAB ARAP Proteins Are Crucial for Autophagosome-Lysosome Fusion But Not Autophagosome Formation During PINK1/Parkin Mitophagy and Starvation. J Cell Biol (2016) 215:857–74. doi: 10.1083/jcb.201607039 PMC5166504 27864321

[42] Sun A Wei J Childress C Shaw JH Peng K Shao G. The E3 Ubiquitin Ligase NEDD4 is an LC3-Interactive Protein and Regulates Autophagy. Autophagy (2017) 13:522–37. doi: 10.1080/15548627.2016.1268301 PMC5361608 28085563

[43] Rambold AS Pearce EL. Mitochondrial Dynamics at the Interface of Immune Cell Metabolism and Function. Trends Immunol (2018) 39:6–18. doi: 10.1016/j.it.2017.08.006 28923365

[44] Ballabio A Bonifacino JS. Lysosomes as Dynamic Regulators of Cell and Organismal Homeostasis. Nat Rev Mol Cell Biol (2020) 21:101–18. doi: 10.1038/s41580-019-0185-4 31768005

[45] Chen Y Yan R Li B Liu J Liu X Song W. Silencing CCNG1 Protects MPC-5 Cells From High Glucose-Induced Proliferation-Inhibition and Apoptosis-Promotion *via* MDM2/p53 Signaling Pathway. Int Urol Nephrol (2020) 52:581–93. doi: 10.1007/s11255-020-02383-4 32016904

[46] Yoo EG Lee WJ Kim JH Chae HW Hyun SE Kim DH. Insulin-Like Growth Factor-Binding Protein-3 Mediates High Glucose-Induced Apoptosis by Increasing Oxidative Stress in Proximal Tubular Epithelial Cells. Endocrinology (2011) 152:3135–42. doi: 10.1210/en.2010-1122 21652730

[47] Palikaras K Lionaki E Tavernarakis N. Coordination of Mitophagy and Mitochondrial Biogenesis During Ageing in C. Elegans. Nature (2015) 521:525–8. doi: 10.1038/nature14300 25896323

[48] Padman BS Nguyen TN Lazarou M. Autophagosome Formation and Cargo Sequestration in the Absence of LC3/GABARAPs. Autophagy (2017) 13:772–4. doi: 10.1080/15548627.2017.1281492 PMC5388231 28165849

[49] Birts CN Banerjee A Darley M Dunlop CR Nelson S Nijjar SK. P53 Is Regulated by Aerobic Glycolysis in Cancer Cells by the CtBP Family of NADH-Dependent Transcriptional Regulators. Sci Signaling (2020) 13:1–29. doi: 10.1126/scisignal.aaz1854 PMC7244340 32371497

