# Supplementary material for: Transcriptome Analysis Reveal Candidate Genes and Pathways Responses to Lactate Dehydrogenase Inhibition (Oxamate) in Hyperglycemic Human Renal Proximal Epithelial Tubular Cells
Source: Front Endocrinol (Lausanne). 2022 Mar 17;13:785605. doi: 10.3389/fendo.2022.785605 (PMC8970056; doi:10.3389/fendo.2022.785605)
Supplement: Supplementary Figure 1 — R2 of the free-scale topology and mean connectivity with soft threshold (power) for 20,377 genes. [file DataSheet_1.zip › Supplementary Figures 1 and 2.docx]

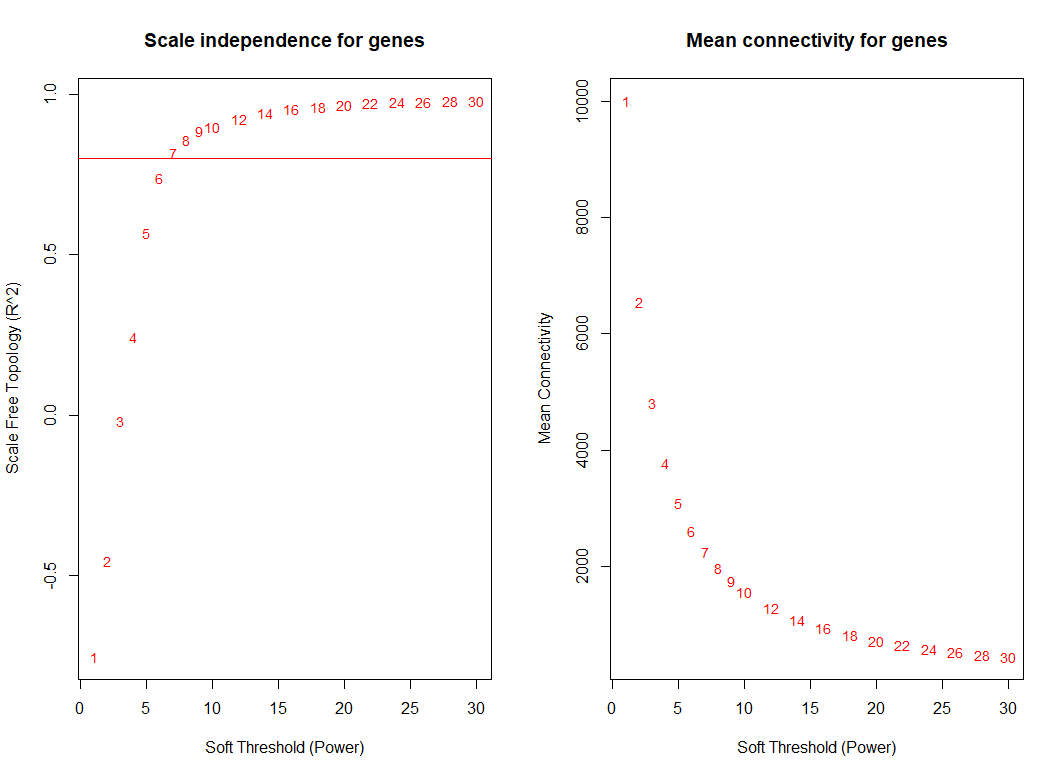
Supplementary figure 1. R^2^ of the free-scale topology and mean connectivity with soft threshold (power) for genes.


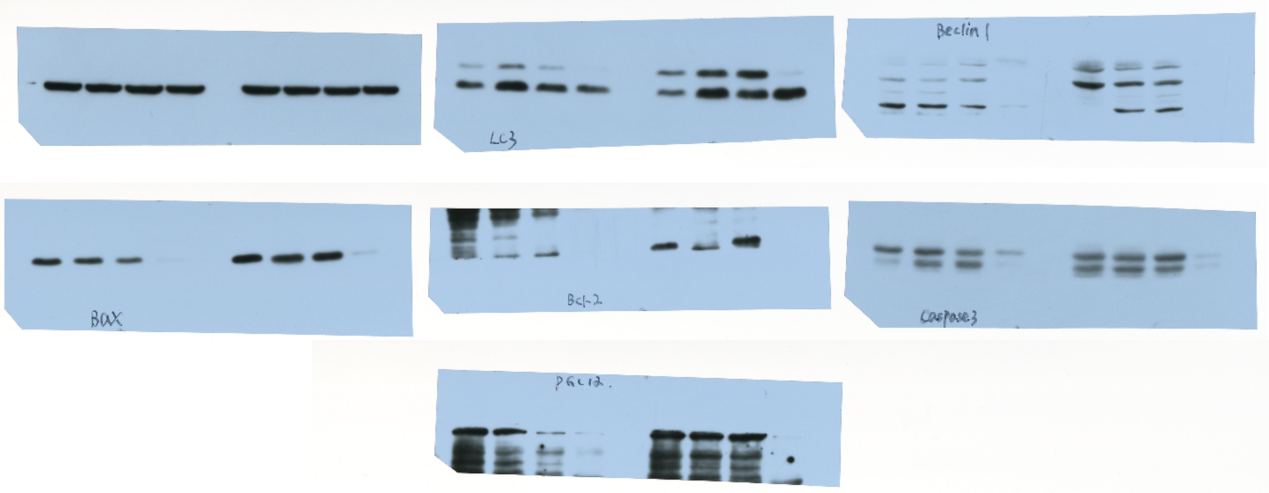


Supplementary figure 2. The original Western blot gels of protein levels of peroxisome proliferator-activated receptor-g coactivator 1α (PGC1α), caspase 3 (CASP3), B-cell lymphoma 2 apoptosis regulator (BCL2), BCL2 associated X apoptosis regulator (BAX), beclin1 (BECN1) and microtubule-associated proteins 1A/1B light chain 3 (MAP1LC3) in HK-2 cells treated with low D-glucose (LG) with different concentrations of oxamate (LGOXA-0mM, LGOXA-20mM, LGOXA-40mM and LGOXA-80mM) and high D-glucose (HG) with different concentrations of oxamate (HGOXA-0mM, HGOXA-20mM, HGOXA-40mM and HGOXA-80mM) for 24 hours.
